# Effect of the Reverse Cotton Osteotomy on First Metatarsal Position: A Cadaveric Study

**DOI:** 10.1177/10711007261438452

**Published:** 2026-04-29

**Authors:** Rowan Urquhart, William Mayer, Hirbod Abootalebi, Aneisha Taunque, Erin Bigney, Oreoluwa Alugo, Chris A. McGibbon, Jacob Matz

**Affiliations:** 1 Faculty of Medicine, Dalhousie Medicine New Brunswick, Saint John, NB, Canada; 2 Canada East Foot & Ankle, Saint John, NB, Canada; 3 Department of Medical Neuroscience, Dalhousie University, Halifax, NS, Canada; 4 Canada East Spine Centre, Saint John, NB, Canada; 5 Horizon Health Network, Saint John, NB, Canada; 6 Faculty of Kinesiology, University of New Brunswick, Fredericton, NB, Canada; 7 Institute of Biomedical Engineering, University of New Brunswick, Fredericton, NB, Canada; 8 Department of Orthopedic Surgery, Horizon Health Network, Saint John, NB, Canada

**Keywords:** pes planus, reconstruction, osteotomy, medial column, forefoot varus

## Abstract

**Background:**

Flatfoot deformities are characterized by collapse of the medial arch due to tendinous and ligamentous dysfunction. The degree of deformity can be evaluated radiographically by measuring the talo–first metatarsal angle (Meary angle). These deformities have traditionally been corrected using a medial cuneiform dorsal opening wedge (Cotton) osteotomy; however, this procedure requires bone grafting to fill the osteotomy site and results in lengthening of the medial column. To address these concerns, the plantar closing wedge of the medial cuneiform bone, known as the reverse Cotton osteotomy (RCO), was developed. In this study, we aim to determine the effect of the RCO on the position of the first metatarsal, thus providing guidance and recommendations for surgeons employing the reverse Cotton osteotomy in the correction of adult-acquired flatfoot.

**Methods:**

In this cadaveric study, we characterized the mean angular correction achieved by 1-, 2-, 3-, and 4-mm RCO by radiographically measuring Meary angle under simulated weight-bearing conditions. Secondary outcomes included radiographic measurements of the navicular-cuneiform incongruency angle (NCIA) and calcaneal pitch angle.

**Results:**

Statistically significant increases in Meary angle were observed following 1-mm (*P* = .020), 2-mm (*P* = .049), and 3-mm (*P* = .049) osteotomies but not following the 4-mm osteotomy (*P* = .081). The mean angular correction achieved by the 1-, 2-, and 3-mm wedge sizes was 0.97°/mm. With the numbers available, no significant increase in NCIA could be detected following any of the RCO wedge sizes, and calcaneal pitch increased significantly only after 2-mm RCO (*P* = .036).

**Conclusion:**

The RCO was associated with significant (*P* ≤ .049) plantarflexion of the first ray in a cadaveric model; however, the authors recommend limiting the wedge size to a maximum of 3 mm, as larger wedges were not associated with further angular correction of the medial arch.

**Clinical Relevance:**

The findings of this study can guide surgeons performing RCO to correct flatfoot deformities, thereby potentially contributing to improved postoperative outcomes.

## Graphical abstract

**Visual abstract:**
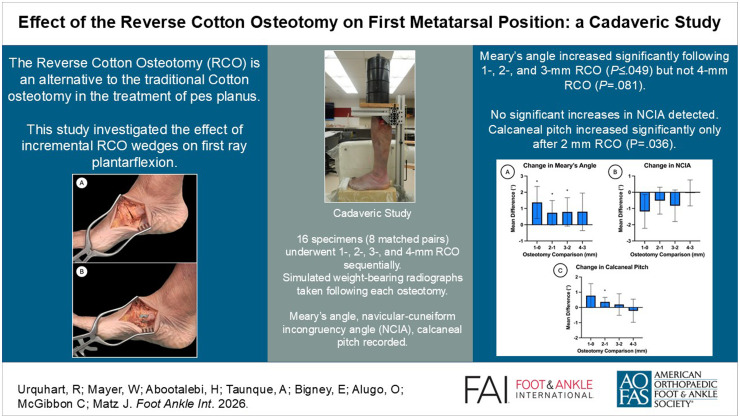
This is a visual representation of the abstract.

## Introduction

Adult-acquired flatfoot deformity is a common condition treated by orthopaedic surgeons, the pathophysiology of which is related to a variety of ligamentous and tendinous injuries. Most commonly, it is associated with posterior tibial tendon dysfunction (PTTD) resulting in a loss of oppositional forces to the pull of the fibularis brevis and longus, consequently leading to hindfoot valgus deformity and collapse of the medial arch.^1-3^ The resulting deformity and medial column instability characterizes adult-acquired flatfoot deformity and, as such, these deformities are typically treated by combining multiple bony realignment procedures. Although hindfoot procedures such as medializing calcaneal osteotomies have been employed to address hindfoot valgus by restoring the mechanical axis of the leg,^4^ residual forefoot varus must additionally be corrected by increasing the degree of first-ray plantarflexion via medial column procedures such as the dorsal opening wedge (Cotton) osteotomy of the medial cuneiform bone.^1,3,5^

Although the Cotton osteotomy effectively achieves plantarflexion of the first metatarsal,^6^ concerns include the need for bone graft to fill the osteotomy and the requisite lengthening of the medial column, which may affect union rates and lead to abduction of the forefoot, respectively.^7^ Therefore, a plantar closing wedge osteotomy of the medial cuneiform bone, referred to as the reverse Cotton osteotomy (RCO),^8^ has been proposed to be advantageous by achieving deformity correction via shortening of the medial column and by eliminating the need for bone graft.^8^

Unfortunately, the angular correction achieved by the RCO has yet to be characterized, representing an important gap in the literature. The objective of this study is to describe the impact of sequentially sized RCO wedges on the talo–first metatarsal angle (Meary angle) and associated radiographic parameters.

## Methods

### Specimens

Sixteen clinical-grade cadaveric specimens (8 matched pairs) obtained from the Dalhousie Body Donation program were used in this study. The total sample size complies with recommendations for cadaveric research, considering factors such as ethical regulations and human donor availability.^9^ All samples were below-knee specimens prepared with a light-embalming technique that uses a reduced-fixative base, humectants, salts, and antimicrobial agents to maintain elasticity, pliability, and tissue responsiveness, similar to living tissue—the Sandeski^10^ technique (formerly Halifax Clinical Cadaver Preparation)—and stored at 4 °C. Our sample was composed of 6 males and 2 females with a mean age of 79.4 years (SD = 8.4). All 8 donors were White. This study was approved by the Horizon Health Network Research Ethics Board.

### Procedure

**Figure 1.**
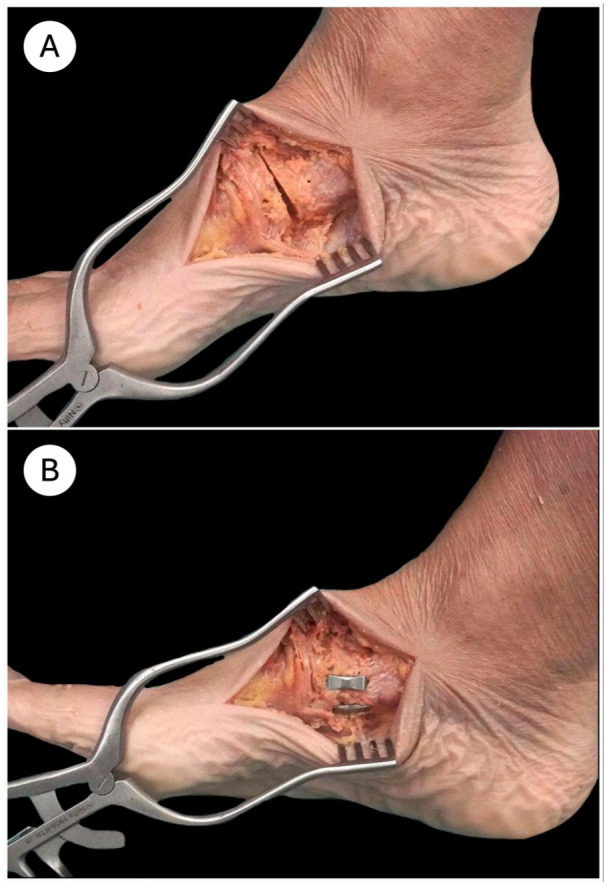
(A) A cadaveric specimen is shown with a 3-mm reverse Cotton osteotomy visualized through a medial incision. (B) The osteotomy is compressed and fixed with two 15-mm compression staples placed medially and plantarly.

The operation performed was a plantar closing wedge osteotomy of the medial cuneiform bone.^8^ All surgeries were performed by a fellowship-trained orthopaedic foot and ankle surgeon and co-author. Exposure of the medial cuneiform was achieved through a 5-cm longitudinal medial incision, taking care to protect the tibialis anterior tendon and avoiding disruption of the plantar stabilizing ligaments of the talonavicular and naviculocuneiform joints. A sagittal saw (Hall^®^ MicroFree^®^ Sagittal Saw XE; ConMed) was introduced to the plantar-medial aspect of the medial cuneiform and the osteotomy was carried out, ensuring to preserve the dorsal cortex, before manually closing the wedge and fixing the osteotomy site with compression staples (BME SPEED Continuous Compression Implant System; DePuy Synthes). The osteotomy along with fixation is demonstrated in Figure 1.

### Loading Conditions

Each specimen was imaged under simulated weight-bearing conditions at baseline and then subsequently following each incremental osteotomy. These loading conditions were achieved using a custom-built rig that allowed fixation of the specimen and subsequent application of constant axial loading of a known magnitude (304 N) to the proximal aspect of the tibia. The proximal fibula was trimmed pre-operatively to ensure complete transmission of the load through the tibia. The rig consisted of an aluminum tower fixed to a wooden base, with a sliding load support platform inserted into the vertically oriented sliding tracks of the aluminum tower and a 31-kg load stack resting on the sliding support platform (Supplementary Figure S1). This setup allowed standardized loading of the leg and ankle, ensuring consistency between specimens.

Under simulated weight-bearing conditions, postoperative perfect lateral radiographs were obtained, determined by a single line outlining the talar dome. The staples were then removed, and the osteotomy was subsequently enlarged in successive 1-mm increments up to a maximum wedge size of 4 mm, and simulated weight-bearing radiographs were repeated following each incremental osteotomy.

### Measures and Statistical Analysis

**Figure 2.**
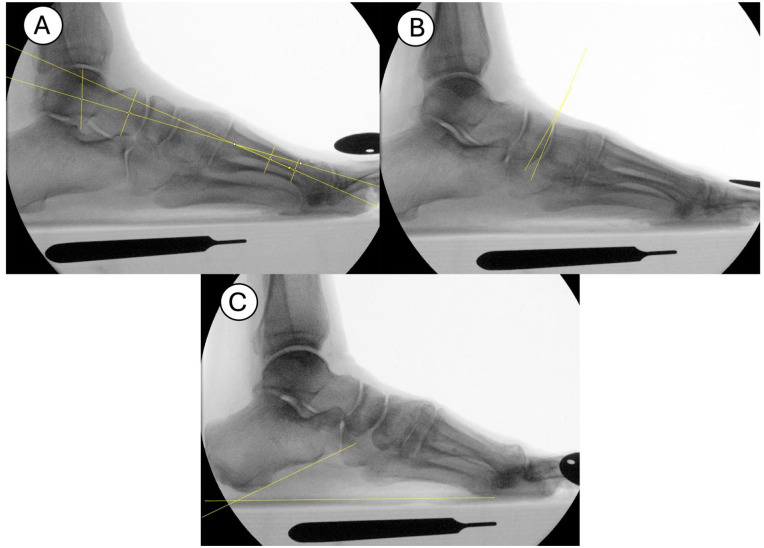
Radiographic measurements of (A) Meary angle, (B) NCIA, and (C) calcaneal pitch angle obtained using perfect lateral plain film radiographs of the foot. (A) The long axis of the talus is drawn through the centre of the talar body and the centre of the talar neck, and the long axis of the first metatarsal is drawn through the centre of the metatarsal shaft. (B) A line is drawn connecting the dorsal and plantar apices of the articular surface of both the navicular and cuneiform sides of the cuneonavicular joint. (C) A line is drawn parallel to the supporting surface of the foot, and a second line is drawn from the plantar cortex of the calcaneal tuberosity to the plantar cortex of the calcaneal side of the calcaneocuboid joint. NCIA, navicular-cuneiform incongruency angle.

Meary angle was measured radiographically under simulated weight-bearing conditions on lateral radiographs pre-operatively and following each osteotomy for each specimen. The value was obtained by intersecting 2 lines: the first bisecting the mid-talar axis and the second bisecting the longitudinal axis of the first metatarsal (Figure 2A). Apex dorsal Meary angle was defined as negative.^11^

Secondary outcomes included the navicular-cuneiform incongruency angle (NCIA) and the calcaneal pitch angle. The NCIA measurement was obtained by drawing one line through the dorsal and plantar aspects of the articular surface of the navicular, and a second line through the dorsal and plantar aspects of the articular surface of the medial cuneiform and measuring the angle of their intersection^12^ (Figure 2B). Apex dorsal NCIA was defined as negative. The calcaneal pitch angle was measured radiographically by drawing a first horizontal line parallel to the supporting surface of the calcaneus and the metatarsal heads, and a second line extending from the plantar cortex of the calcaneal component of the calcaneocuboid joint to the plantar cortex of the calcaneal tuberosity (Figure 2C).

Two independent raters measured Meary angle, NCIA, and calcaneal pitch angle for each osteotomy size. Within each osteotomy size, the scores reported by the 2 raters were averaged to obtain single mean value for each radiographic parameter. These mean values were then compared using paired *t* tests with a Benjamini-Hochberg correction for multiple comparisons, with the level of significance set at *P <*.05. Inter-rater reliability was assessed using a 2-way mixed effects model for averaged measures to evaluate absolute agreement between the 2 raters. Statistical analyses were conducted using IBM SPSS Statistics version 29.0.2.0 (20).^13^ As a measure of overall cadaveric study quality, Biomechanics Objective Basic Science Quality Assessment Tool (BOBQAT) scores (0-100 points)^14^ were calculated by 2 independent raters, and the mean of the 2 scores was reported.

## Results

**Figure 3.**
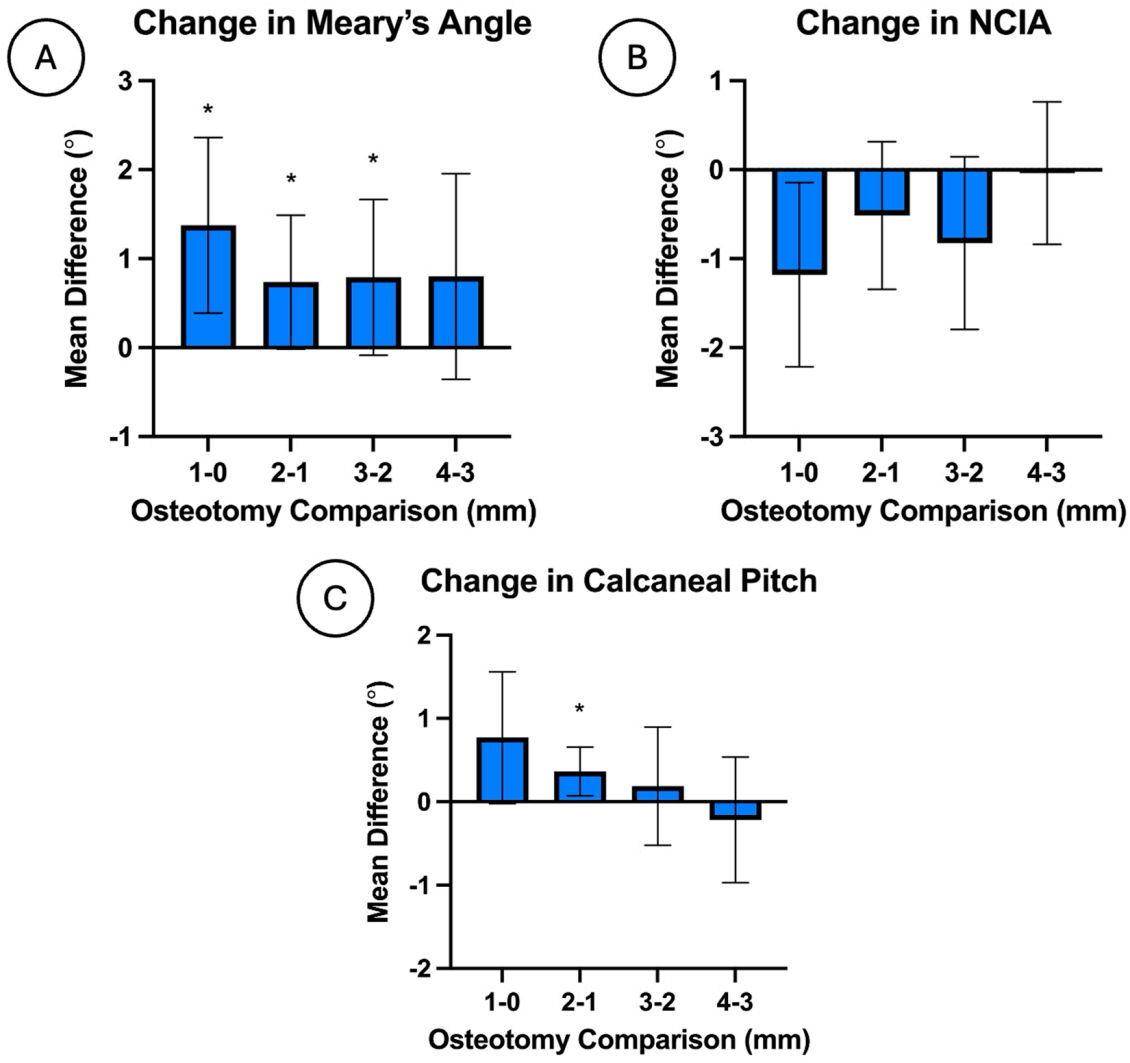
Bar charts visualizing the mean differences in (A) Meary angle, (B) NCIA, and (C) calcaneal pitch between sequential RCO wedges. The mean difference is represented in degrees. Each bar represents one comparison, and the wedge sizes in millimeters being compared are represented on the X axis. The significance of each comparison is shown (*P < .5). Significance was determined with paired *t* tests and a Benjamini-Hochberg correction to control the false discovery rate. NCIA, navicular-cuneiform incongruency angle; RCO, reverse Cotton osteotomy.

The anatomy of the medial cuneiform was characterized by a mean length of 23.03 mm (SD = 1.65) and a mean height of 30.24 mm (SD = 2.57). The mean preoperative Meary angle was 5.87°. Meary angle increased significantly by 1.38° following 1-mm RCO (*P* = .020), 0.74° following 2-mm RCO (*P* = .049), and 0.79° following 3-mm RCO (*P* = .049). With the numbers available, no significant increase in Meary angle could be detected following 4-mm RCO (*P* = .081) (Figure 3). For Meary angle, inter-rater reliability ranged from good to excellent across osteotomy sizes (Table 1). The overall mean change in Meary angle per millimeter increase in osteotomy size up to 3 mm was 0.97°/mm.

**Table 1.** Summary of Descriptive Statistics for 3 Radiographic Parameters (Meary Angle, NCIA, Calcaneal Pitch) by RCO Wedge Size.^a^

Wedge Size (mm)	Meary Angle (°), Mean ± SD [95% CI]	NCIA (°), Mean ± SD [95% CI]	Calcaneal Pitch (°), Mean ± SD [95% CI]
0 baseline	5.46 ± 6.65 [1.92, 9.01]	−5.05 ± 2.56 [−6.41, −3.69]	23.35 ± 4.89 [21.07, 26.35]
ICC [95% CI]	0.92 [0.77, 0.97]	0.79 [0.41, 0.93]	0.98 [0.93, 0.99]
1 mm	7.98 ± 7.38 [4.05, 11.92]	−7.01 ± 2.58 [−8.39, −5.64]	24.32 ± 5.40 [21.44, 27.20]
ICC [95% CI]	0.95 [0.83, 0.98]	0.50 [−0.20, 0.81]	0.99 [0.96, 0.99]
2 mm	9.29 ± 7.26 [5.31, 13.26]	−7.88 ± 1.64 [−8.76, −7.01]	24.42 ± 5.45 [21.51, 27.32]
ICC [95% CI]	0.93 [0.63, 0.98]	0.28 [−0.26, 0.68]	0.99 [0.97, 0.99]
3 mm	10.69 ± 7.26 [6.82, 14.56]	−8.68 ± 2.48 [−10.00, −7.35]	24.65 ± 5.49 [21.73, 27.58]
ICC [95% CI]	0.87 [0.06, 0.97]	0.47 [−0.24, 0.80]	0.98 [0.93, 0.99]
4 mm	10.92 ± 7.79 [6.77, 15.07]	−8.77 ± 2.76 [−10.24, −7.30]	24.08 ± 5.35 [21.22, 26.93]
ICC [95% CI]	0.89 [0.60, 0.96]	0.67 [−0.21, 0.90]	0.98 [0.92, 0.99]

Abbreviations: ICC, intraclass correlation coefficient; NCIA, navicular-cuneiform incongruency angle; RCO, reverse Cotton osteotomy.

a ICCs were calculated using a 2-way mixed effects model (average measures, absolute agreement).

The mean preoperative NCIA was −4.84°, and with the numbers available, no significant increase could be detected following any subsequent osteotomies (Figure 3). Inter-rater reliability for NCIA ranged from poor to good (Table 1). The mean preoperative calcaneal pitch angle was 23.35°. Calcaneal pitch only increased significantly after 2-mm RCO (*P* = .036; Figure 3). Inter-rater reliability for calcaneal pitch was excellent across all osteotomy sizes (Table 1). Descriptive statistics are summarized in Table 1 and group comparisons are summarized in Table 2. The mean BOBQAT score was 82.5/100.

**Table 2.** Summary of Mean Differences in Meary Angle, NCIA, and Calcaneal Pitch Between RCO Wedge Sizes and the Corresponding Paired *t* Tests.^a^

	Meary Angle		NCIA		Calcaneal Pitch	
Wedge Comparison (mm)	Mean Difference ± SD [95% CI]	Adjusted *P*	Mean Difference ± SD [95% CI]	Adjusted *P*	Mean Difference ± SD [95% CI]	Adjusted *P*
1-0	1.38 ± 1.85 [0.39, 2.36]	**.020**	−1.18 ± 1.94 [−2.22, −0.15]	.056	0.77 ± 1.49 [−0.02, 1.56]	.056
2-1	0.74 ± 1.41 [−0.02, 1.49]	**.049**	−0.51 ± 1.56 [−1.34, 0.32]	.137	0.37 ± 0.55 [0.07, 0.66]	**.036**
3-2	0.79 ± 1.64 [−0.08, 1.66]	**.049**	−0.825 ± 1.82 [−1.80, 0.15]	.090	0.19 ± 1.33 [−0.52, 0.90]	.291
4-3	0.80 ± 2.17 [−0.36, 1.96]	.081	−0.04 ± 1.50 [−0.84, 0.76]	.458	−0.22 ± 1.42 [−0.97, 0.54]	.291

Abbreviations: NCIA, navicular-cuneiform incongruency angle; RCO, reverse Cotton osteotomy.

a The wedge comparison column depicts the RCO wedge sizes in millimeters that were compared with paired *t* tests, where 0 represents baseline. Adjusted *P* values were obtained with a Benjamini-Hochberg correction to control the false discovery rate. Boldface represents statistically significant *P* values (<.05).

## Discussion

The reverse Cotton osteotomy is one tool in the arsenal of surgeons treating adult acquired pes planus that is most often employed in combination with other bony realignment procedures, with the objective of increasing first metatarsal plantarflexion and restoring the positioning of the first ray in the Cotton triangle of support.^1,8^ It is because of this capacity to plantarflex the first metatarsal that medial column procedures are frequently necessary to correct residual forefoot varus; lateral column lengthening procedures in isolation may correct forefoot abduction whereas varus deformity often persists,^5^ resulting in lateral column overloading and lateral midfoot pain.^15,16^

This study demonstrates that the RCO procedure achieves, on average, an increase in Meary angle of 0.97°/mm increase in wedge size. This is nearly 1° less per millimeter than the degree of angular correction achieved by the Cotton osteotomy in a previous report by Scott and colleagues that found a mean correction of 1.9°/mm ± 0.2°/mm increment in wedge size.^17^ Interestingly, the angular correction achieved by the RCO in the current study was limited to a maximum wedge size of 3 mm, whereas Scott and colleagues found continual correction up to a wedge size of 6.5 mm with the Cotton osteotomy.^17^ A limitation of the study by Scott et al was the loading parameters, as the specimens were loaded inconsistently and generally underloaded, with loads ranging from 20 to 30 lb, which may partly explain the contrast in findings. Other studies investigating simulated weight-bearing parameters with fixed loads have suggested the use of partial weight-bearing loads of 35 kg.^18,19^

There are several factors likely contributing to the observed limitations of the corrective power of the RCO. Firstly, although the observed increases in NCIA in the present study were not statistically significant, the upward trend suggests that the corrective capacity of the RCO may be partly limited by compensatory extension at the naviculocuneiform joint with increasing plantarflexion of the first metatarsal under loaded conditions. Additionally, employing larger closing wedge osteotomies is accompanied by requisite increases in loss of bone stock. In this study, the mean length of the medial cuneiform bone was approximately 23 mm, which is in line with the findings of a recent computed tomography–based study of medial cuneiform anatomy that determined the mean length of the medial cuneiform to be 24 ± 2.4 mm.^20^ Thus, executing a 4-mm closing wedge osteotomy results in the loss of approximately 17% of the total bone length on average. This considerable bone loss coupled with the requisite usage of larger, more robust compression staples in the fixation of large RCO wedge sizes likely results in compromised structural integrity of the cancellous bone, especially under loaded conditions. Although the authors recognize that pes planus reconstruction post-operative protocols typically entail strict non–weight-bearing for 6-8 weeks, it is nonetheless important to consider the theoretically increased risk of implant failure associated with large RCO wedge sizes and the associated post-operative complications in clinical practice. Moreover, the inter-rater reliability for the NCIA measurements ranged from poor to good because of the position of the hardware obscuring the articular margins on lateral radiographs, particularly in cases with larger wedges removed. This further supports the concern for compromised bone integrity associated with larger wedge sizes and raises the potential for intra-articular fracture because of the proximity of the implants to the joint.

Although the mean BOBQAT score indicates good overall cadaveric study quality, this study has several limitations. Firstly, we had a sample size of 16 specimens (8 matched pairs) which, although similar to other cadaveric studies, is nonetheless small and limits the statistical power of the study. These specimens additionally had an advanced mean age of 79.4 years, meaning the sample was largely composed of specimens with osteopenic bone. It is therefore possible that larger RCO wedge sizes may be more feasible in younger individuals with more bone density. Furthermore, this sample was composed exclusively of White specimens, which limits the generalizability of these findings to the general population. Finally, the use of cadaveric specimens requires reliance on the static stabilizers of the foot and ankle because of the absence of muscular tonus and normal dynamic forces present during weight-bearing in living individuals. As such, further documentation of clinical outcomes of the RCO in living individuals would be beneficial.

## Conclusion

In this study, we discovered that the reverse Cotton osteotomy is effective in achieving plantarflexion of the first metatarsal as measured by Meary angle. We recommend that RCO wedge size be limited to a maximum of 3 mm because of the consequent loss of bone stock and potential risk of implant failure, combined with diminished angular correction with wedges beyond 3 mm. Based on these cadaveric findings, the RCO procedure may be of limited use in cases of pes planus with residual forefoot varus requiring 5 or more degrees of correction of Meary angle.

## Supplemental Material

sj-jpg-2-fai-10.1177_10711007261438452 – Supplemental material for Effect of the Reverse Cotton Osteotomy on First Metatarsal Position: A Cadaveric Study Supplemental material, sj-jpg-2-fai-10.1177_10711007261438452 for Effect of the Reverse Cotton Osteotomy on First Metatarsal Position: A Cadaveric Study by Rowan Urquhart, William Mayer, Hirbod Abootalebi, Aneisha Taunque, Erin Bigney, Oreoluwa Alugo, Chris A. McGibbon and Jacob Matz in Foot & Ankle International

sj-pdf-1-fai-10.1177_10711007261438452 – Supplemental material for Effect of the Reverse Cotton Osteotomy on First Metatarsal Position: A Cadaveric Study Supplemental material, sj-pdf-1-fai-10.1177_10711007261438452 for Effect of the Reverse Cotton Osteotomy on First Metatarsal Position: A Cadaveric Study by Rowan Urquhart, William Mayer, Hirbod Abootalebi, Aneisha Taunque, Erin Bigney, Oreoluwa Alugo, Chris A. McGibbon and Jacob Matz in Foot & Ankle International
