# Supplementary figures and images for: Effect of the Reverse Cotton Osteotomy on First Metatarsal Position: A Cadaveric Study
Source: Foot Ankle Int. 2026 Apr 29;47(7):918–24. doi: 10.1177/10711007261438452 (PMC13365300; doi:10.1177/10711007261438452)

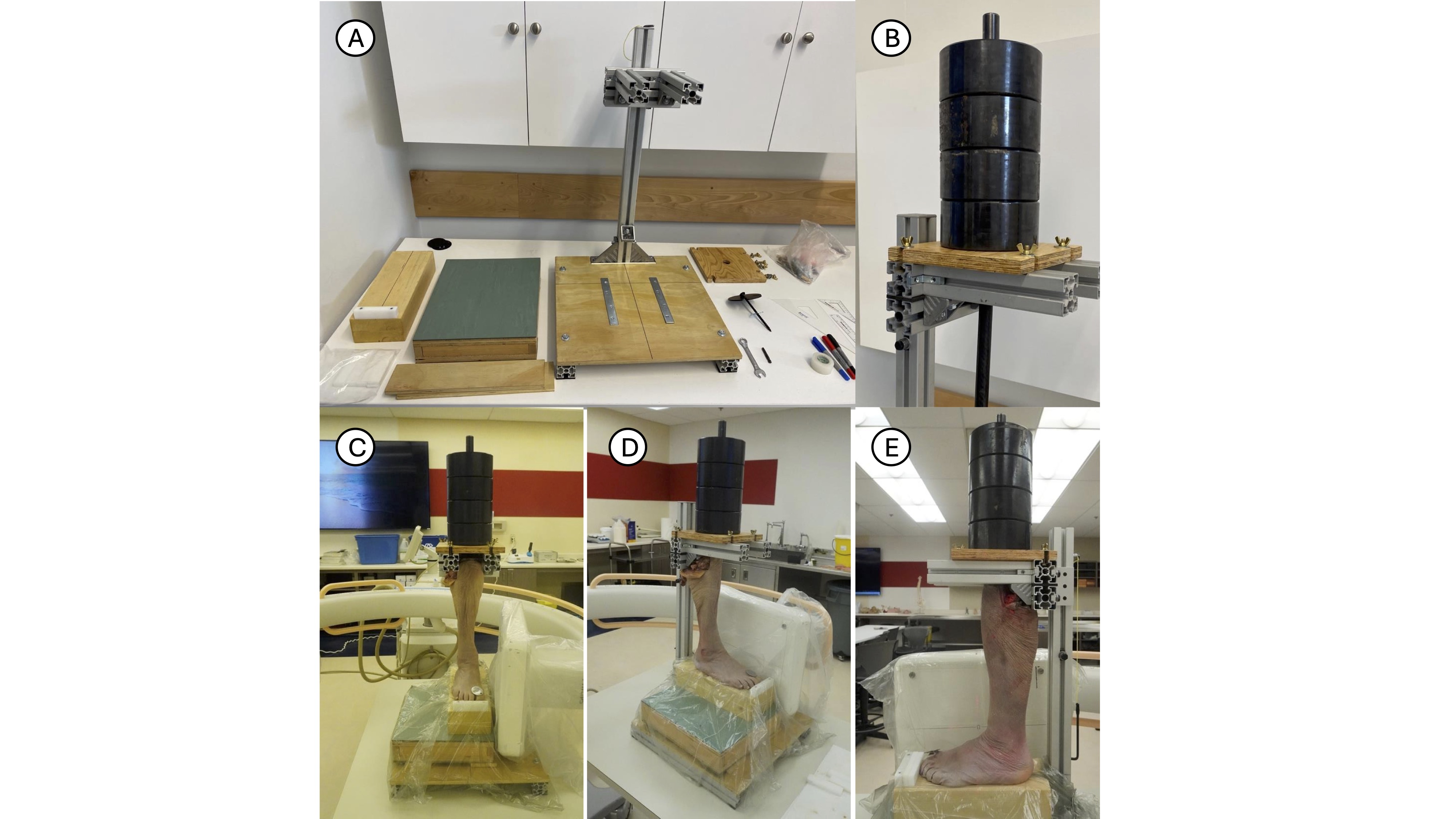

Supplement: sj-jpg-2-fai-10.1177_10711007261438452 – Supplemental material for Effect of the Reverse Cotton Osteotomy on First Metatarsal Position: A Cadaveric Study [file sj-jpg-2-fai-10.1177_10711007261438452.jpg]
